# Validity and responsiveness of EuroQol-5 dimension (EQ-5D) versus Short Form-6 dimension (SF-6D) questionnaire in chronic pain

**DOI:** 10.1186/1477-7525-11-110

**Published:** 2013-07-01

**Authors:** Marko Obradovic, Arun Lal, Hiltrud Liedgens

**Affiliations:** 1 Grunenthal GmbH, Zieglerstrasse 6, Aachen 52099, Germany

**Keywords:** Quality of life, Chronic pain, Tapentadol, Utility, Health technology assessment, Cost effectiveness, Cost utility, Health economics

## Abstract

**Background:**

Assessments of health-related quality of life and particularly utility values are important components of health economic analyses. Several instruments have been developed to measure utilities. However no consensus has emerged regarding the most appropriate instrument within a therapeutic area such as chronic pain. The study compared two instruments – EQ-5D and SF-6D – for their performance and validity in patients with chronic pain.

**Methods:**

Pooled data from three randomised, controlled clinical trials with two active treatment groups were used. The included patients suffered from osteoarthritis knee pain or low back pain. Differences between the utility measures were compared in terms of mean values at baseline and endpoint, Bland–Altman analysis, correlation between the dimensions, construct validity, and responsiveness.

**Results:**

The analysis included 1977 patients, most with severe pain on the Numeric Rating Scale. The EQ-5D showed a greater mean change from baseline to endpoint compared with the SF-6D (0.43 to 0.58 versus 0.59 to 0.64). Bland–Altman analysis suggested the difference between two measures depended on the health status of a patient. Spearmans rank correlation showed moderate correlation between EQ-5D and SF-6D dimensions. Construct validity showed both instruments could differentiate between patient subgroups with different severities of adverse events and analgesic efficacies but larger differences were detected with the EQ-5D. Similarly, when anchoring the measures to a disease-specific questionnaire – Western Ontario and McMaster Universities Osteoarthritis Index (WOMAC) – both questionnaires could differentiate between WOMAC severity levels but the EQ-5D showed greater differences. Responsiveness was also higher with the EQ-5D and for the subgroups in which improvements in health status were expected or when WOMAC severity level was reduced the improvements with EQ-5D were higher than with SF-6D.

**Conclusions:**

This analysis showed that the mean EQ-5D scores were lower than mean SF-6D scores in patients with chronic pain. EQ-5D seemed to have higher construct validity and responsiveness in these patients.

## Background

Chronic pain represents a major health burden in Europe affecting as many as one in five adults [1-3] Pain can be directly related to absenteeism and unemployment, and severe daily pain has been shown to increase healthcare resource utilization with increased visits to healthcare providers, including hospitalisations [4-6]. It also has a major impact on an individual’s quality of life [6] which can substantially decrease with increasing pain severity [7]. Many patients with chronic pain report being less able, or no longer able, to take part in various daily activities, such as walking, driving, working outside home, sleeping, etc. [2].

The impact of both illness and treatment of illness on quality of life is increasingly measured using quality adjusted life years (QALYs) and ‘utility’ scores that represent people’s preferences towards a particular health state. A utility is a metric used in health economic evaluations to capture quality of life and is used as a basis of cost-utility analysis, the most common type of health economic evaluation used in health technology assessment. Utilities are measured on an interval scale from zero to one; zero reflects states of health equivalent to death and one reflects full health. The utility score is based on the definition and description of a set of health states together with the valuation of those health states according to the strength of preference for each. Direct measurement of utilities can be undertaken for any health state. The two classical direct measurement techniques are the standard gamble (SG) and the time trade-off (TTO). The SG estimates utility of a particular health state based on the maximum risk of immediate death that an individual is willing to accept in order to gain full health. The TTO estimates utility based on the life time an individual is willing to give up for a shorter period in full health. Thus, there is a systematic difference in utilities derived from SG and TTO. However, these methods are time-consuming, complex and at times, may not be ethical [8]. As a result, indirect measurement of utilities is more commonly undertaken by using pre-scored, multi-attribute health status classification systems [9]. These generic instruments include the EuroQol-5 dimension (EQ-5D), Short Form-6 dimension (SF-6D), and Health Utilities Index 3 (HUI3) and generate utilities that can be used to compare QALYs across different patient groups and diseases.

The EQ-5D, SF-6D and HUI3 differ considerably in terms of their dimensions, items and preference weights [8] and there is no consensus on which of these is best or most useful in individual conditions including in chronic pain. The EQ-5D tends to be the measure most used in cost-utility analysis and in health technology assessment; for example the use of QALYs is required by the National Institute for Health and Clinical Excellence (NICE) in the UK with the EQ-5D as the preferred measure of utility [10]. Alternative measures can be used if there is evidence that EQ-5D is not suitable for a particular patient group [10].

The EQ-5D is a standardised instrument for use as a measure of health outcome developed by the EurolQol group, a consortium of investigators in Europe [11]. It is applicable to a variety of different illnesses and treatments and provides a simple descriptive profile and a single index value for health status. The five dimensions included in the EQ-5D are: mobility; self-care; usual activities; pain/discomfort and anxiety/depression. Each dimension has three levels (no problems, some problems, major problems) and together defines 243 health states (3 to the power of 5 gives the 243 possible combination), to which has been added “unconscious” and “dead” for a total of 245 health states. There are a number of country-specific scoring functions available which allow derivation of utilities specific to certain settings. In the UK, for example, a utility score is assigned to each health state based on the preferences elicited from a survey of 3395 adults by using the time trade-off method [12]. EQ-5D scores range between −0.594 and 1 (full health).

The SF-6D is derived from the health-related quality of life questionnaire, the Short Form 36 [13]. It has six dimensions: physical functioning; role limitations; social functioning; pain; mental health and vitality; the classification system consists of four to six levels on each of the six attributes, giving a total of 18,000 health states. The SF-6D comes with a set of preference weights obtained from a sample of the general population in the UK using the standard gamble valuation technique [13].

Here we report an analysis undertaken to compare the performance and validity of EQ-5D versus SF-6D in patients with chronic pain due to osteoarthritis of the knee or low back pain based on data from three similar phase III trials [14]. The two measures were possible to compare because the EQ-5D and SF-36 (from which SF-6D is derived) were both used in these studies.

## Methods

This study analysed data pooled from three randomised, controlled studies [14]. All three trials had similar design; randomised, multicentre, double-blind, parallel-group, placebo-controlled, and active controlled. Eligible patients included both men and women with a clinical diagnosis of either osteoarthritis knee pain (two studies; ClinicalTrials.gov Identifiers NCT00421928 [15], and NCT00486811) or non-malignant low back pain (one study; ClinicalTrials.gov Identifier NCT0044917615 [16]). Patients had been taking analgesics for the pain for at least three months, and were dissatisfied with their current analgesic therapy. The inclusion criteria mandated patients to have an average pain intensity score at baseline of ≥5 on an 11-point numerical rating scale (NRS; 0 = ‘no pain’ to 10 = ‘pain as bad as you can imagine‘). Patients received twice daily doses of placebo, tapentadol PR (100–250 mg), or oxycodone HCl CR (20–50 mg) for a 12-week maintenance period, preceded by a 3-week titration period. Primary endpoints were change from baseline in average pain intensity at week 12 of the maintenance period and for the overall maintenance period. Secondary endpoints included the SF-36 health survey and the EQ-5D health survey.

This analysis combined data from all the patients in the two active treatment groups and compared the mean values of the EQ-5D and SF-6D at baseline and endpoint, as well as exploring differences between the two measures using a Bland–Altman plot. This is a statistical method that uses a graphical presentation to illustrate how differences between two measures depend on the health status of a patient [17]. Combined data were also used to compare distributions across severity levels of the EQ-5D and SF-6D dimensions and correlations across dimensions using Spearman’s rank coefficient.

Construct validity was undertaken to test the ability of each instrument to distinguish between known subgroups. In one analysis the ability of the two utility measurements to discriminate between health states with different severities of adverse events and analgesic efficacies was tested. A proportion of patients having experienced an adverse event (typically constipation, nausea and vomiting, dizziness, somnolence) discontinued treatment as they found adverse events too bothersome. Some patients discontinued treatment due to lack of efficacy and other patients tolerated the treatment well, having no adverse events and completing the trial. Rates of these events have been reported in Lange et al. 2010 [14]. Therefore, these subgroups represent different severities of adverse events and analgesic efficacies of treatments and can be used to test construct validity of the two questionnaires compared. In another analysis, the ability of each instrument to distinguish among patient subgroups in the two osteoarthritis studies which were shown to differ based on a disease specific measure, i.e. anchoring to the Western Ontario and McMaster Universities Osteoarthritis Index (WOMAC) was analysed. WOMAC is a standardised questionnaire used to assess pain, stiffness, and physical function in patients with hip and / or knee osteoarthritis [18]. The WOMAC measures five items for pain (score range 0–20), two for stiffness (score range 0–8), and 17 for functional limitation (score range 0–68). The total WOMAC score is created by summing the items for all three subscales, with higher scores reflecting worse pain, stiffness, and physical function. Based on the total score patients were classified into four groups with increasing severity level: none to mild (total score of 0 to ≤24), mild to moderate (total score of >24 to ≤48), moderate to severe (total score of >49 to ≤72), and severe to extreme (total score of >72 to ≤96). WOMAC’s reliability and validity have been demonstrated in many countries [19].

The responsiveness or ability to detect improvements (or deterioration) in a health state, of each instrument was also analysed using the standardized response mean (SRM). The SRM is the average difference divided by the standard deviation of the differences between the paired measurements. Improvements in the EQ-5D versus the SF-6D utilities were measured for patients with different severity levels based on the overall WOMAC score at baseline versus study endpoint (for osteoarthritis studies). Improvements in the EQ-5D compared with the SF-6D utilities were also measured for patients with different severities of adverse events and analgesic efficacies.

## Results

The analysis included a total of 1977 patients, the majority of whom had severe pain (Table 1). Mean values for baseline EQ‒5D and SF‒6D utilities compared with mean values for endpoint showed a greater change from baseline to endpoint with EQ-5D compared with SF-6D (0.43 to 0.58 [mean change of 0.15; 95% CI: 0.143 - 0.169] versus 0.59 to 0.64 [mean change of 0.05; 95% CI: 0.045-0.055]). In addition these mean values highlighted differences in scoring at baseline for each measurement scale, with EQ-5D giving lower values.

**Table 1:** Baseline demographics and patient characteristics

**Characteristic**	**Tapentadol prolonged**	**Oxycodone controlled**
	**release (n = 978)**	**release (n = 999)**
Mean ± SD age, years	56.8 ± 12.22	56.7 ± 12.39
Age category, n (%):		
<65 years	719 (73.5)	731 (73.2)
≥65 years	259 (26.5)	268 (26.8)
Gender, n (%)		
Female	639 (65.3)	601 (60.2%)
Male	339 (34.7)	398 (39.8)
Race, n (%)		
White	803 (82.1)	813 (81.4)
Black	111 (11.3)	101 (10.1)
Hispanic	40 (4.1)	58 (5.8)
Other	24 (2.5)	27 (2.7)
Pain condition, n (%)		
Osteoarthritis knee pain	663 (67.8)	673 (67.4)
Low back pain	315 (32.2)	326 (32.6)
Mean ± SD pain intensity score *+	7.4 ± 1.26	7.3 ± 1.21
Pain intensity category, n (%) + ~		
Mild	2 (0.2)	0
Moderate	119 (12.2)	123 (12.3)
Severe	854 (87.6)	873 (87.7)
Prior opioid experience, n (%)		
No	641 (65.5)	681 (68.2)
Yes	337 (34.5)	318 (31.8)

*Average pain score (11 point numerical rating scale) recorded over the last 72 hours prior to randomisation.

+ Tapentadol n = 975, oxycodone n = 996.

~ Mild pain intensity defined as 1 to <4 on the numerical rating scale, moderate >4 to <6 and severe ≥6.

**Figure 1:**
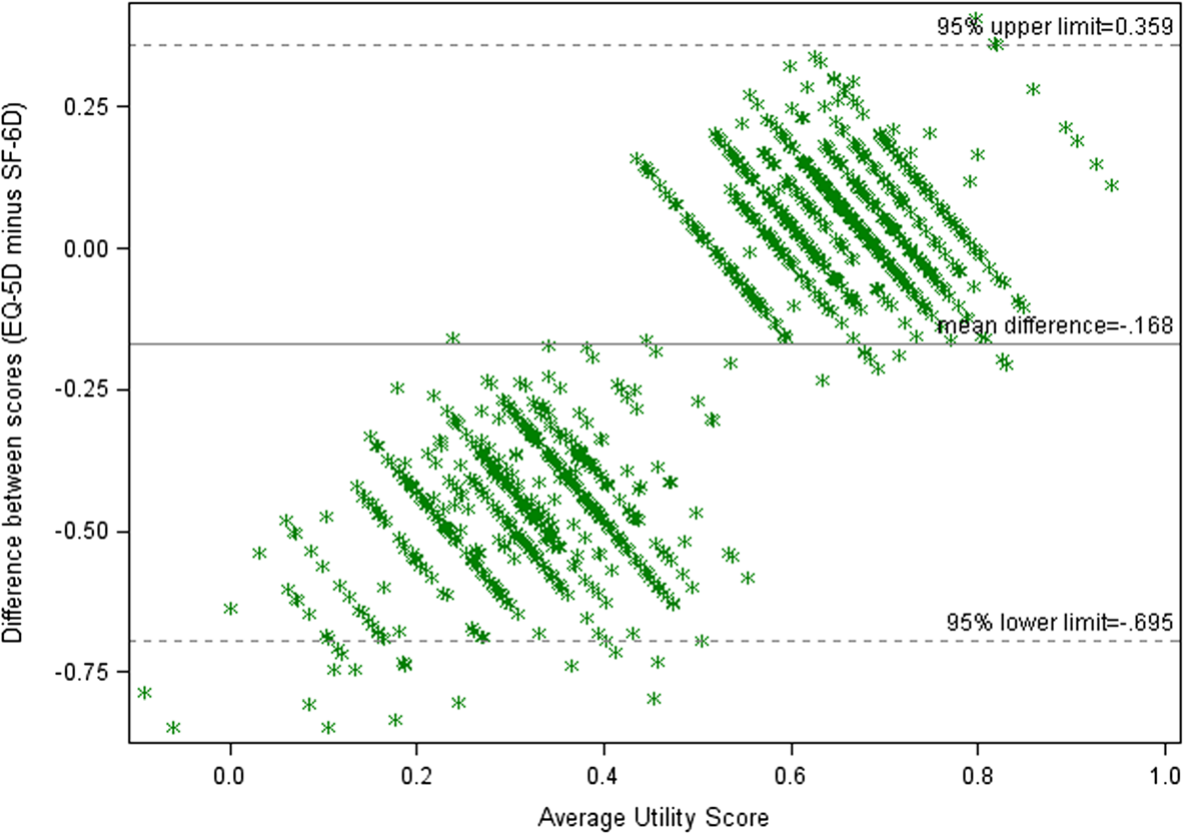
Bland Altman plot for EQ-5D and SF-6D scores at baseline.

The Bland–Altman plot for assessment of agreement of the two methods of measurement suggested that the differences between the two measurements depended on the health status of the individual patient. Patients with low quality of life (average utility <0.6) had lower scores on the EQ-5D and, conversely, those with high quality of life (average utility >0.8) had greater scores on the EQ-5D. However, for those patients with mid-range utility values, the EQ-5D and SF-6D were more aligned (Figure 1).

**Figure 2:**
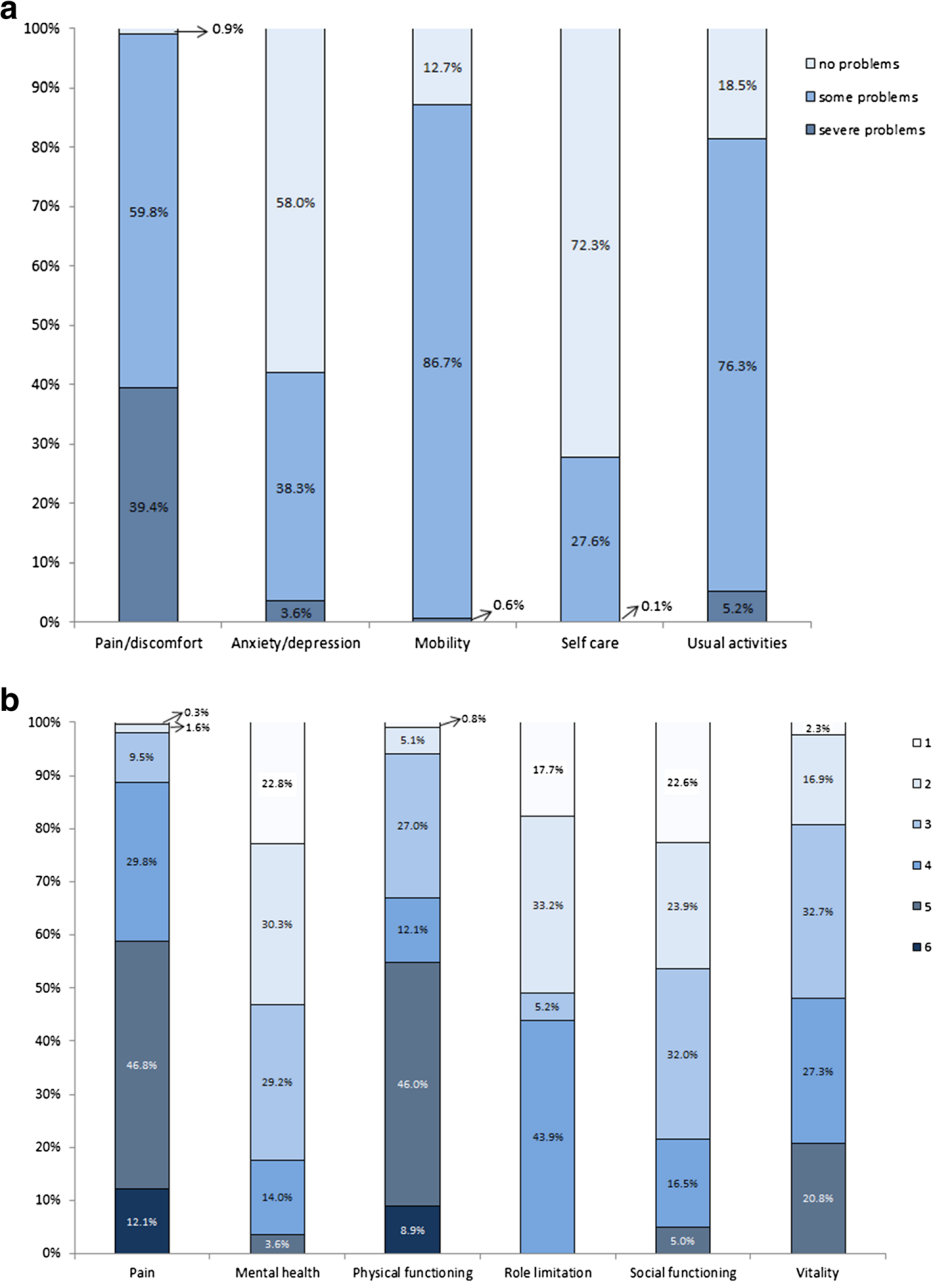
**Distribution across severity levels of the EQ-5D and SF-6D dimensions at baseline. (a)** Distribution across severity levels of the EQ-5D dimensions at baseline. **(b)** Distribution across severity levels of the SF-6D dimensions at baseline.

In EQ-5D patients most frequently noted having severe problems in “pain/discomfort” dimension and no problems in “self care” and “anxiety/depression” dimensions (Figure 2a). In SF-6D the most severe problems were seen in “pain” and “physical functioning” dimensions and least problems in “mental health”, “role limitation” and “social functioning” (Figure 2b). Analysis of correlation between EQ-5D and SF-6D dimensions revealed a moderate correlation. Among the similar dimensions, there was stronger correlation between pain and pain/discomfort; mental health and anxiety/depression; physical functioning and self-care; role limitation and usual activities; social functioning and anxiety/depression (Table 2). Vitality exists only for the SF-6D and not surprisingly, therefore, it exhibited a weaker correlation with EQ-5D dimensions.

**Table 2:** Correlation between EQ-5D and SF-6D dimensions (using Spearman’s rank coefficient)

		**SF-6D dimensions**					
		**Pain**	**Mental health**	**Physical functioning**	**Role limitation**	**Social functioning**	**Vitality**
**EQ-5D dimensions**	**Pain/discomfort**	**0.46**	0.11	0.22	0.18	0.25	0.21
	**Anxiety/depression**	0.24	**0.51**	0.20	0.21	**0.40**	0.20
	**Mobility**	0.23	0.10	0.24	0.19	0.18	0.10
	**Self care**	0.22	0.21	**0.41**	0.17	0.29	0.13
	**Usual activities**	0.37	0.13	0.29	**0.030**	0.30	0.20

Construct validity suggested that both questionnaires could differentiate between patient subgroups in terms of tolerating the treatment, having adverse events, discontinuing due to adverse events and discontinuing due to lack of efficacy. Differences between subgroups were larger in case of EQ-5D (Table 3). Additionally, both questionnaires could differentiate between WOMAC severity levels; the mean EQ-5D and SF-6D values decreased with increasing WOMAC severity levels which is in line with expectations. The differences between EQ-5D and SF-6D increased with increasing WOMAC severity and showed the greatest difference in the severe to extreme group (Table 3). The differences between EQ-5D and SF-6D for more severe WOMAC states were substantially larger than the minimal important differences (MID) of both questionnaires.

**Table 3:** Construct validity: comparison of EQ-5D and SF-6D in terms of patient subgroup differentiation and WOMAC severity level (at baseline) differentiation

	**EQ-5D**	**SF-6D**	**Difference**
			**(EQ-5D vs. SF-6D)**
**Patient subgroups**			
Patients who completed the trial and had no adverse events	0.695	0.694	0.001
Patients with at least one AE	0.583	0.640	−0.057
Patients with an AE that lead to withdrawal	0.503	0.594	−0.091
Patients who discontinued therapy due to lack of efficacy	0.405	0.582	−0.176
**WOMAC severity level at baseline**			
None to mild	0.740	0.717	0.023
Mild to moderate	0.550	0.616	−0.066
Moderate to severe	0.311	0.536	−0.225
Severe to extreme	0.180	0.461	−0.281

**Figure 3:**
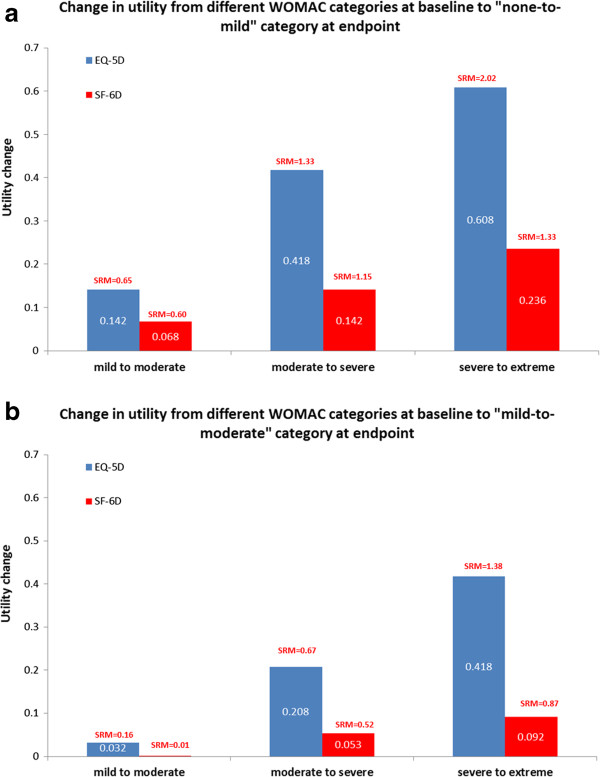
**Comparison of EQ-5D and SF-6D in terms of responsiveness. (a)** Change in utility from different WOMAC categories at baseline to ‘none to mild’ at endpoint. **(b)** Change in utility from different WOMAC categories at baseline to ‘mild-to-moderate’ at endpoint.

Responsiveness analysis suggested that improvements in EQ-5D versus SF-6D utilities were higher with EQ-5D. The EQ-5D had a higher standardized response mean, especially for patients in the ‘severe to extreme’ level of overall WOMAC severity at baseline (Figure 3). For the subgroups where there were expected improvements in health status (patients who completed the trial and had no adverse events or patients who completed the trial and had >30% pain relief), the EQ-5D improvements were higher than SF-6D improvements. This was also seen for other subgroups; those experiencing adverse events, patients discontinuing due to adverse events and patients discontinuing due to lack of efficacy. For patients who discontinued therapy either due to adverse events or lack of efficacy, the SF-6D showed no change, whereas a slight improvement was seen with the EQ-5D (Table 4).

**Table 4:** Comparison of EQ-5D and SF-6D in patient subgroups where there were expected improvements in health status

**Patient subgroup**	**EQ-5D change from baseline**	**SF-6D change from baseline**
Patients who completed the trial and had no adverse events	0.252	0.094
Patients who completed the trial and had >30% pain relief	0.286	0.113
Patients with at least one AE	0.157	0.049
Patients with an AE that lead to withdrawal	0.073	0.005
Patients who discontinued therapy due to lack of efficacy	0.032	0.006

## Discussion

This study represents one of the larger analyses undertaken to date comparing the EQ-5D and SF-6D and suggests that the EQ-5D may be the more appropriate technique for measurement of utility values in severe chronic pain.

The results of this analysis suggested that the mean EQ-5D scores were generally lower than mean SF-6D scores in chronic pain and that the difference between these two measures was dependent on the quality of life status of individual patients. The differences in mean utilities between patients with different severity levels based on a disease-specific questionnaire (WOMAC) were substantially larger with the EQ-5D compared with the SF-6D. In addition, the EQ-5D had higher responsiveness; improvements utilities for patients with different severity levels were higher with the EQ-5D. It also detected larger differences between various health states with different adverse events, severity and pain relief, and produced larger changes in utility from baseline to endpoint for these groups.

Similar results have been found in other studies, both in patients with pain [20-23] and in other illnesses [24,25]. In a cross-sectional study of 275 individuals with chronic low back pain, Søgaard and colleagues found that the mean value of EQ-5D was significantly lower than that of the SF-6D, whereas the variation across observations was significantly greater for the EQ-5D [20]. They also found that the difference between measures was associated with the average value of health, i.e., the poorer the health, the higher the SF-6D overestimation relative to EQ-5D. In a survey of 389 patients with knee pain, Barton *et al.* compared the EQ-5D and SF-6D for practicality, validity and responsiveness [21]. The results suggested that while the EQ-5D and SF-6D had largely comparable practicality and construct validity, the SF-6D could not discriminate between those who improved post-intervention, and those who did not. In another study in patients with knee pain due to inflammatory arthritis [22], the EQ-5D was found to be more responsive to deterioration in health, whereas the SF-6D was more responsive to improvement in health. The results showed that the SF-6D did not respond well to deterioration in patients with established severe rheumatoid arthritis, suggesting the SF-6D may be inappropriate for patients with severe progressive disease. Comparison of the EQ-5D and SF-6D across seven different conditions or patient groups - chronic obstructive airways disease, osteoarthritis, irritable bowel syndrome, lower back pain, leg ulcers, post-menopausal women and the elderly - has highlighted their general agreement in producing roughly similar indices over this large and variable group [24]. However, this same analysis also illustrated the considerable disagreement between these two measures; the intra-class correlation coefficient for the whole sample was 0.51, and depending on the patient group, the difference between the measures ranged from 0.015 to 0.094 and the intra-class correlation coefficient from 0.27 to 0.55.

The differences between EQ-5D and SF-6D are unsurprising since both measures vary in their descriptive systems, number of levels, scoring function, range of utilities and recall period [23,24,26,27]. Both also have differences in the valuation methods used; the EQ-5D has been valued using time trade-off, while the SF-6D is a derivative of the SF-36 and was valued using standard gamble techniques [28].

While differences between these two instruments might be unsurprising, any analyses that aim to compare these different preference based measures of health are important because they inform debate and choice. Whether the discrepancy between utility measures might influence health economic analyses and policy decision-making is not yet clear though some evidence suggests it might [29,30]. In a simulation of over 100,000 patients with rheumatoid arthritis, for example, Marra and colleagues [29] showed that 91% of the simulations favoured the cost utility of a specific treatment when using the HUI3 to calculate QALYs. However, when using the EQ-5D, HUI2, or the SF-6D utility values, the proportion of simulations that favoured the cost utility of the treatment were 63%, 45%, and 12%, respectively. A review by McDonough and colleagues concluded that choice of preference measure may contribute to qualitatively different incremental cost-effectiveness ratios (ICERs) under some circumstances [30]. Specifically, as ICERs rise, the probability of acceptance appears to decrease, making the differences in QALYs obtained using different methods potentially meaningful. This could be particularly important for treatments with long-term consequences and ICERs around common thresholds.

At present the EQ-5D tends to provide a wider scoring range and better completion rates than the SF-6D [23] and tends to provide more favourable cost-effectiveness ratios [30]. A new 5-level version of the EQ-5D has recently been launched, the EQ-5D-5L [31] where the labels for each of the dimensions are: no problems, slight problems, moderate problems, severe problems and unable to/extreme problems. Studies to derive value sets for the EQ-5D-5L are currently under development and the potential usefulness has yet to be fully investigated. However, it is anticipated that this new 5-level version will have better discriminative capacity and sensitivity to change and smaller ceiling effects than the EQ-5D-3L.

## Conclusions

Differences between EQ-5D and SF-6D values can be expected due to intrinsic differences in these indirect measurements of utility. This analysis of patients with chronic pain showed that the mean EQ-5D scores were lower than mean SF-6D scores, however, the difference depended on the quality of life status of a patient. EQ-5D seemed to have higher construct validity and responsiveness in this patient population. Namely, differences in mean utilities between osteoarthritis patients with different severity levels based on a disease-specific questionnaire (WOMAC) were substantially larger with EQ-5D compared to SF-6D. Similarly, EQ-5D detected larger differences between various patient subgroups (including both osteoarthritis and low back pain studies) with different AEs severity and pain relief. Moreover, the measure of responsiveness was numerically higher for EQ-5D, especially for subgroups with low QOL levels at baseline.

## Competing interests

MO, AL and HL are all employees of Grünenthal GmbH.

## Authors' contributions

MO, AL and HL all participated in the design of the study, AL performed the statistical analysis, and MO coordinated the manuscript development. All authors reviewed, contributed and approved the final manuscript.

## References

[B1] Rustoen T Wahl AK Hanestad BR Lerdal A Paul S Miaskowski C Prevalence and characteristics of chronic pain in the general Norwegian population Eur J Pain 2004 8 555 565 10.1016/j.ejpain.2004.02.002 15531224

[B2] Breivik H Collett B Ventafridda V Cohen R Gallacher D Survey of chronic pain in Europe: prevalence, impact on daily life, and treatment Eur J Pain 2006 10 287 333 10.1016/j.ejpain.2005.06.009 16095934

[B3] Sjogren P Ekholm O Peuckmann V Gronbaek M Epidemiology of chronic pain in Denmark: an update Eur J Pain 2009 13 287 292 10.1016/j.ejpain.2008.04.007 18547844

[B4] Langley P Muller-Schwefe G Nicolaou A Liedgens H Pergolizzi J Varrassi G The impact of pain on labor force participation, absenteeism and presenteeism in the European Union J Med Econ 2010 13 662 672 10.3111/13696998.2010.529379 21034378

[B5] Langley P Muller-Schwefe G Nicolaou A Liedgens H Pergolizzi J Varrassi G The societal impact of pain in the European Union: health-related quality of life and healthcare resource utilization J Med Econ 2010 13 571 581 10.3111/13696998.2010.516709 20815688

[B6] Langley PC The prevalence, correlates and treatment of pain in the European Union Curr Med Res Opin 2011 27 463 480 10.1185/03007995.2010.542136 21194390

[B7] McDermott AM Toelle TR Rowbotham DJ Schaefer CP Dukes EM The burden of neuropathic pain: results from a cross-sectional survey Eur J Pain 2006 10 127 135 10.1016/j.ejpain.2005.01.014 16310716

[B8] Whitehead SJ Ali S Health outcomes in economic evaluation: the QALY and utilities Br Med Bull 2010 96 5 21 10.1093/bmb/ldq033 21037243

[B9] Drummond MF Sculpher MJ Torrance GW O'Brien BJ Stoddart GL Methods for the evaluation of health care programmes 2005 Third edition Oxford: Oxford University Press

[B10] Brazier J Longworth L NICE DSU technical support document 8: an introduction to the measurement and valuation of health for NICE submissions report by the Decision Support Unit 2011 NICE http://www.nicedsu.org.uk/TSD8%20Introduction%20to%20MVH_final.pdf 28481495

[B11] The EuroQol Group.EuroQol–a EuroQol-a new facility for the measurement of health-related quality of life. The EuroQol Group Health Policy 1990 16 199 208 10109801 10.1016/0168-8510(90)90421-9

[B12] Dolan P Modeling valuations for EuroQol health states Med Care 1997 35 1095 1108 10.1097/00005650-199711000-00002 9366889

[B13] Brazier J Roberts J Deverill M The estimation of a preference-based measure of health from the SF-36 J Health Econ 2002 21 271 292 10.1016/S0167-6296(01)00130-8 11939242

[B14] Lange B Kuperwasser B Okamoto A Steup A Haufel T Ashworth J Etropolski M Efficacy and safety of tapentadol prolonged release for chronic osteoarthritis pain and low back pain Adv Ther 2010 27 381 399 10.1007/s12325-010-0036-3 20556560

[B15] Afilalo M Etropolski MS Kuperwasser B Kelly K Okamoto A Van HI Steup A Lange B Rauschkolb C Haeussler J Efficacy and safety of Tapentadol extended release compared with oxycodone controlled release for the management of moderate to severe chronic pain related to osteoarthritis of the knee: a randomized, double-blind, placebo- and active-controlled phase III study Clin Drug Investig 2010 30 489 505 10.2165/11533440-000000000-00000 20586515

[B16] Buynak R Shapiro DY Okamoto A Van HI Rauschkolb C Steup A Lange B Lange C Etropolski M Efficacy and safety of tapentadol extended release for the management of chronic low back pain: results of a prospective, randomized, double-blind, placebo- and active-controlled Phase III study Expert Opin Pharmacother 2010 11 1787 1804 10.1517/14656566.2010.497720 20578811

[B17] Bland JM Altman DG Statistical methods for assessing agreement between two methods of clinical measurement Lancet 1986 1 307 310 2868172

[B18] Bellamy N Buchanan WW Goldsmith CH Campbell J Stitt LW Validation study of WOMAC: a health status instrument for measuring clinically important patient relevant outcomes to antirheumatic drug therapy in patients with osteoarthritis of the hip or knee J Rheumatol 1988 15 1833 1840 3068365

[B19] Bellamy N WOMAC: a 20-year experiential review of a patient-centered self-reported health status questionnaire J Rheumatol 2002 29 2473 2476 12465137

[B20] Sogaard R Christensen FB Videbaek TS Bunger C Christiansen T Interchangeability of the EQ-5D and the SF-6D in long-lasting low back pain Value Health 2009 12 606 612 10.1111/j.1524-4733.2008.00466.x 19900258

[B21] Barton GR Sach TH Avery AJ Doherty M Jenkinson C Muir KR Comparing the performance of the EQ-5D and SF-6D when measuring the benefits of alleviating knee pain Cost Eff Resour Alloc 2009 7 12 10.1186/1478-7547-7-12 19615052 PMC2720915

[B22] Harrison MJ Davies LM Bansback NJ McCoy MJ Verstappen SM Watson K Symmons DP The comparative responsiveness of the EQ-5D and SF-6D to change in patients with inflammatory arthritis Qual Life Res 2009 18 1195 1205 10.1007/s11136-009-9539-2 19777373 PMC2761817

[B23] Whitehurst DG Bryan S Another study showing that two preference-based measures of health-related quality of life (EQ-5D and SF-6D) are not interchangeable. But why should we expect them to be? Value Health 2011 14 531 538 10.1016/j.jval.2010.09.002 21315635

[B24] Brazier J Roberts J Tsuchiya A Busschbach J A comparison of the EQ-5D and SF-6D across seven patient groups Health Econ 2004 13 873 884 10.1002/hec.866 15362179

[B25] Longworth L Bryan S An empirical comparison of EQ-5D and SF-6D in liver transplant patients Health Econ 2004 12 1061 1067 14673814 10.1002/hec.787

[B26] Bryan S Longworth L Measuring health-related utility: why the disparity between EQ-5D and SF-6D? Eur J Health Econ 2005 6 253 260 10.1007/s10198-005-0299-9 15968563

[B27] Grieve R Grishchenko M Cairns J SF-6D versus EQ-5D: reasons for differences in utility scores and impact on reported cost-utility Eur J Health Econ 2009 10 15 23 10.1007/s10198-008-0097-2 18327677

[B28] Tsuchiya A Brazier J Roberts J Comparison of valuation methods used to generate the EQ-5D and the SF-6D value sets J Health Econ 2006 25 334 346 10.1016/j.jhealeco.2005.09.003 16271783

[B29] Marra CA Marion SA Guh DP Najafzadeh M Wolfe F Esdaile JM Clarke AE Gignac MA Anis AH Not all "quality-adjusted life years" are equal J Clin Epidemiol 2007 60 616 624 10.1016/j.jclinepi.2006.09.006 17493521

[B30] McDonough CM Tosteson AN Measuring preferences for cost-utility analysis: how choice of method may influence decision-making Pharmaco Economics 2007 25 93 106 10.2165/00019053-200725020-00003 PMC3046553 17249853

[B31] Herdman M Gudex C Lloyd A Janssen M Kind P Parkin D Bonsel G Badia X Development and preliminary testing of the new five-level version of EQ-5D (EQ-5D-5L) Qual Life Res 2011 20 1727 1736 10.1007/s11136-011-9903-x 21479777 PMC3220807

